# Physiologically based pharmacokinetic modeling of tea catechin mixture in rats and humans

**DOI:** 10.1002/prp2.305

**Published:** 2017-04-17

**Authors:** Francis C. P. Law, Meicun Yao, Hui‐Chang Bi, Stephen Lam

**Affiliations:** ^1^ Department of Biological SciencesSimon Fraser University8888 University DriveBurnabyBritish ColumbiaCanada; ^2^ School of Pharmaceutical SciencesSun Yat‐sen UniversityGuangzhouGuangdongChina; ^3^ Departments of Respiratory MedicinePathology and Cancer ImagingBritish Columbia Cancer Agency, and the University of British ColumbiaVancouverBritish ColumbiaCanada

**Keywords:** PBPK model, tea catechins, systemic dosimetry

## Abstract

Although green tea (*Camellia* sinensis) (GT) contains a large number of polyphenolic compounds with anti‐oxidative and anti‐proliferative activities, little is known of the pharmacokinetics and tissue dose of tea catechins (TCs) as a chemical mixture in humans. The objectives of this study were to develop and validate a physiologically based pharmacokinetic (PBPK) model of tea catechin mixture (TCM) in rats and humans, and to predict an integrated or total concentration of TCM in the plasma of humans after consuming GT or Polyphenon E (PE). To this end, a PBPK model of epigallocatechin gallate (EGCg) consisting of 13 first‐order, blood flow‐limited tissue compartments was first developed in rats. The rat model was scaled up to humans by replacing its physiological parameters, pharmacokinetic parameters and tissue/blood partition coefficients (PCs) with human‐specific values. Both rat and human EGCg models were then extrapolated to other TCs by substituting its physicochemical parameters, pharmacokinetic parameters, and PCs with catechin‐specific values. Finally, a PBPK model of TCM was constructed by linking three rat (or human) tea catechin models together without including a description for pharmacokinetic interaction between the TCs. The mixture PBPK model accurately predicted the pharmacokinetic behaviors of three individual TCs in the plasma of rats and humans after GT or PE consumption. Model‐predicted total TCM concentration in the plasma was linearly related to the dose consumed by humans. The mixture PBPK model is able to translate an external dose of TCM into internal target tissue doses for future safety assessment and dose‐response analysis studies in humans. The modeling framework as described in this paper is also applicable to the bioactive chemical in other plant‐based health products.

**Abbreviations**
AUC: area under the concentration‐time curve
BLPLR: blood/plasma ratio
BW: body weight
CBA: catechin in arterial blood
CBN: catechin in bone
CBR: catechin in brain
CBV: catechin in venous blood
C_expti_: experimental concentration
CFT: catechin in adipose tissue
CGT: catechin in gut tissue
CHR: catechin in heart
CKD: catechin in kidney
CL: clearance
CLG: catechin in lung
CLV: catechin in liver
C_max_: peak plasma concentration of tea catechin
CMS: catechin in muscle
CO: cardiac output
C_predi_: model‐predicted concentration
CRB: catechin in rest of the body
CSK: catechin in skin
CSP: catechin in spleen
EC: epicatechin
ECg: epicatechin gallate
EGC: epigallocatechin
EGCg: epigallocatechin gallate
EROD: ethoxyresorufin‐*O*‐deethylase
*F*: bioavailability factor
*f*_up_: the fraction unbound in plasma
*f*_ut_: the fraction unbound in tissue
GT: Green tea
HPLC: high performance liquid chromatograph
IEF: the inhibitory equivalence factor
*k*_a_, *k*_*r*a_, and *k*_*f*_: respectively are absorption, re‐absorption, and fecal excretion rate constants
LOD: limit of detection
LSP: Log‐normalized sensitivity parameter
MAPE: Mean absolute prediction error
PBPK: physiologically based pharmacokinetic
PCs: partition coefficients
PE: Polyphenon E
PKs: pharmacokinetics
*P*_O:W_: log octanol:water partition coefficient
*P*_VO:W_: log vegetable oil:water partition coefficient
*R*_t_: residence time of catechin in bile
TCM: tea catechin mixture
TCs: tea catechins
*tlag*: absorption lag time
V_n_: fractional weight of neutral fat
V_ph_, fractional weight of phospholipids
V_w_: fractional weight of water

## Introduction

**Figure 1:**
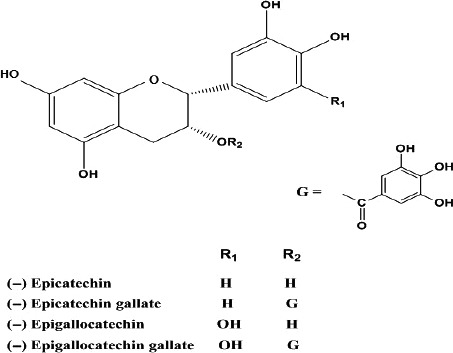
Chemical structures of EGCg, EGC, ECg, and EC.

Green tea (GT), the water extract of *Camellia* sinensis leaves, consists of a complex mixture of tea catechins (TCs) such as epigallocatechin gallate (EGCg), epigallocatechin (EGC), epicatechin gallate (ECg), and epicatechin (EC) (Fig. 1). Daily consumption of GT is believed to be beneficial to health including prevention of cancer, obesity, diabetes, and cardiovascular diseases (Saito et al. 2009). Thus, Polyphenon E (PE), a standardized GT extract, has been found an effective agent in slowing down the progression of early stage cancer in humans (Shanafelt et al. 2013). Also, a PE ointment has been approved by the US Food and Drug Administration for the treatment of genital warts. However, TCs may cause toxic effects in animals and humans especially when they are administered at high doses, that is, 10–29 mg/kg/day tea‐based diets (Lambert et al. 2007).

The pharmacokinetics (PKs) and tissue distribution of TCs have been studied in different animal species including rodents (Chen et al. 1997; Suganuma et al. 1998; Zhu et al. 2000; Cai et al. 2002), dogs (Swezey et al. 2003), and humans (Pietta et al. 1998; Chow et al. 2001; Van Amelsvoort et al. 2001; Lee et al. 2002; Meng et al. 2002). EGCg is the most studied tea catechin because it is the most abundant and potent catechin of GT (Balentine et al. 1997). EGCg is absorbed rapidly by rodents (Kim et al. 2000); peak plasma concentration (*C*_max_) usually appears within 3 h after consumption (Chen et al. 1997; Suganuma et al. 1998). About 0.17% and 6% of the consumed EGCg is eliminated in the urine (Zhu et al. 2001) and bile (Kohri et al. 2001a) of rats, respectively. Less than 0.02% and >90% of the EGCg consumed by humans is excreted in the urine (Van Amelsvoort et al. 2001) and feces (Lee et al. 2002), respectively. TCs are metabolized mainly by conjugation with glucuronic acid and sulfate in rats and humans (Kohri et al. 2001a; Meng et al. 2002; Chow et al. 2005) before being excreted into the bile (Kida et al. 2000). The conjugated metabolites may be converted back to the free forms by intestinal bacteria before undergoing entero‐hepatic recycling (Kohri et al. 2001b). Small amounts of TCs also are metabolized by CYP1A2 (Obermeier et al. 1995) and catechol‐*O*‐methyltransferase (Meng et al. 2002) enzymes. Non‐gallated TCs (e.g., EGC and EC) are more extensively metabolized than gallated TCs (e.g., EGCg and ECg) in rats and humans. The clearance (CL/F) of EGCg, ECG, and EC is 0.061, 0.091, and 0.046 L/min/kg, respectively, in rats (Zhu et al. 2000) but ranges from 0.092 to 0.24 L/min/kg in humans (Chow et al. 2001).

The target organs of GT chemoprevention have been identified in rodents (Yang et al. 2002). However, little is known of the PKs and target organs of a tea catechin mixture (TCM) in humans due to the difficulties and high costs of collecting tissue/organ samples from humans directly. Andersen (1987) has suggested using the physiologically based pharmacokinetic (PBPK) model (IPCS, 2010) to predict the PKs and tissue doses of environmental chemicals in humans. However, only a few PBPK models have been developed for botanical drugs which include soy isoflavone (Schlosser et al. 2006; Law 2007), matrine (Gao and Law 2009), caffeine (Ginsberg et al. 2004), sophoridine (Hu and Huang 1995), and glycyrrhizic acid (Ploeger et al. 2000). Moreover, none of these models are able to predict the PKs and tissue doses of the whole plant in humans because they have been developed with laboratory animals using single marker chemicals of these plants.

The objectives of this study were to develop a PBPK model of TCM for rats and humans after consuming GT or PE, to validate the mixture PBPK model by comparing model simulation with observed data in the literature, and to predict an effect‐based or integrated concentration of TCM in the plasma of humans using *C*_max_ as the dose surrogate (Andersen 1987; Ito et al. 2004). In the present study, the plasma was used as an illustrative example to predict TCM dose metrics in other organs/tissues. Results of the study showed the mixture PBPK model was able to duplicate the kinetic data of three major tea catechin constituents in the plasma of rats and humans after GT/PE consumption. Moreover, total TCM concentrations in the plasma were related linearly to the dose administered to humans. The modeling approach as described in this paper also is applicable to the bioactive chemical mixtures in other plant‐based natural health products such as traditional medicines, functional foods, and dietary supplements.

## Materials and Methods

### Sources of experimental data

The empirical data used to develop and validate the PBPK models were taken from previously published pharmacokinetic studies in rats and humans after receiving an oral dose of pure tea catechin or GT/PE formulation. Since we were unable to obtain the original data of these studies, the observed data were read digitally from the publications using DigiMatic^®^ (Windows version 2.2c, FEB Software. Chesterfield, Virginia). The following was a brief summary of the pharmacokinetic studies:

#### Pharmacokinetic studies in rats

Zhu et al. (2000) studied the PKs of TCs in male Sprague‐Dawley rats (210–230 g) equipped with implanted jugular vein cannuli. Each rat was given an oral dose of PE containing a mixture of EGCg (2500 mg/kg), ECg (650 mg/kg), and EC (250 mg/kg). Blood samples were removed from the jugular cannuli of rats at different time points post‐dosing. The blood samples were centrifuged to separate the plasma from red blood cells. The plasma samples were analyzed for free TCs using a high performance liquid chromatograph (HPLC). Tea catechin concentrations in the plasma samples were plotted against sampling times. The resulting concentration‐time curves were analyzed by the noncompartmental approach. Oral bioavailability of EGCg, ECg, and EC was found to be 0.14, 0.06, and 0.39, respectively; urinary recovery was respectively, 0.17%, 0.25%, and 4.72% of the administered tea catechin doses.

Chen et al. (1997) studied the PKs of TCs in male Sprague‐Dawley rats (310 g) after administering an oral dose of pure EGCg (75 mg/kg) or PE containing a mixture of EGCg (14.6 mg/kg), EGC (13.6 mg/kg) and EC (5.4 mg/kg). Blood samples were removed from the orbital sinus of rats at different time points post‐dosing. The blood samples were centrifuged to separate the plasma from red blood cells. The plasma samples were incubated separately with glucuronidase/sulfatase enzymes at 37°C. The reaction mixture was extracted by ethyl acetate. Total EGCg, EGC or EC concentrations (free catechin plus conjugated metabolites) in ethyl acetate extracts were determined using HPLC and plotted against sampling time. The resulting concentration‐time curves were fitted to the one‐compartment, classical pharmacokinetic model. Results of the study showed the PKs of pure EGCg and crude EGCg were different in the plasma of rats.

#### Pharmacokinetic studies in humans

Chow et al. (2003) studied the PKs of EGCg in eight healthy human volunteers (72 kg) diagnosed with Fitzpatric type II or III skin problems. Each participant was given an oral dose of pure EGCg (400 mg). Blood samples were collected from the volunteers at 0.5, 1.0, 2.0, 3.5, 5.0, 6.5, 8.0, and 24.0 h post‐dosing and centrifuged to separate the plasma from red blood cells. The plasma samples were extracted by ethyl acetate which was analyzed for free EGCg using an ESA Model 580 HPLC equipped with an ESA 5600 Coulochem electrode array system. The plasma concentration‐time curves were analyzed using the non‐compartment approach (WinNonlin version 2.0. Pharsight Corporation, Cary, N.C. USA).

Chow et al. (2001) studied the PKs of EGCg in human volunteers (75 kg) receiving an oral dose of pure EGCg (400 mg) or a PE preparation containing 400 mg of crude EGCg. Blood samples were collected from the volunteers at different time points post‐dosing and centrifuged to separate the plasma from red blood cells. The plasma samples were extracted by ethyl acetate and quantified using a HPLC. The PK profiles of pure EGCg and crude EGCg in PE were found to be very similar. In a separate study, the PKs of EGCg, EGC, and EC were examined simultaneously in the plasma of humans (72 kg) after consuming PE containing a mixture of EGCg (600 mg), EGC (111 mg), and EC (93 mg). Unchanged EGCg, EGC, and EC in the plasma were determined using a HPLC. Only free or unchanged EGCg, and mainly EGC and EC conjugated metabolites were found in the plasma of humans (Chow et al. 2001). The concentration‐time curves of the TCs were analyzed using the non‐compartmental approach.

Lee et al. (2002) studied the PKs of TCs in the plasma of human volunteers (45–85 kg) after administering an oral dose of pure EGCg (2 mg/kg) or GT solids (20 mg/kg) containing a mixture of EGCg (13.9%), EGC (11.0%), and EC (3.2%). Blood samples were removed from the volunteers at predetermined time points, and centrifuged to separate the plasma from red blood cells. The plasma samples were incubated with *β*‐glucuronidase and sulfatase, extracted and analyzed by HPLC equipped with a Coulochem electrode array detector. Free EGCg, EGC, and EC concentrations in the plasma samples were determined as a percentage of total concentration (free catechin plus conjugated metabolites) but only at 1 h and 5 h post‐dosing time points. The concentration‐time curves were separately fitted to the one‐compartment, classical pharmacokinetic model. Large inter‐individual differences in pharmacokinetic parameters especially those related to the oral absorption of TCs were observed in the study. Results of the study also confirmed that the pharmacokinetic behaviors of pure EGCg and crude EGCg were very similar in humans (Chow et al. 2001).

### Developing PBPK models of individual tea catechins for rats

A PBPK model of EGCg was first developed in rats. The rat EGCg model was then converted to an ECg or EC model by replacing the physicochemical parameters, pharmacokinetic parameters, and tissue/blood partition coefficients (PCs) with values specific for the tea catechin (see Tables 1, 2, 3).

**Table 1:** Physicochemical properties and unbound fractions of tea catechins in the plasma of rats

Parameters^a^	EGCg	ECg	EGC	EC
*P*_O:W_	97.70	468	4.84	3.09
*Dvo:w*	5.13	29.3	0.26	0.16
pK_a_	7.75	7.75	9.54	9.54
*f*_up_ b	0.04	0.03	0.25	0.25
*f*_ut_ c	0.08	0.06	0.40	0.40
BLPLR^d^	0.91	0.99	0.88	0.88

a *P*_O:W,_ log octanol‐water partition coefficient; *Dvo:w*, log vegetable oil‐water partition coefficient; pK_a_, ionization constants. *P*_O:W_; and pK_a_ are obtained using the ACD/I‐Labs online prediction engine (https://ilab.acdlabs.com/); *P*_VO:W_, is calculated from *P*_O:W_ and pKa according to Poulin and Theil (2002).

b *f*_up,_ the fraction unbound in plasma, is taken from Zhu et al. (2001)

c *f*_ut,_ the fraction unbound in tissue, is calculated using the equation, *f*_ut_ = 1/[1 + (((1−*f*_up_)/*f*_up_) ×  0.5)] (Poulin and Theil 2002).

d BLPLR, the blood/plasma ratios of individual tea catechins in rats, are predicted using tissue composition‐based model (Poulin and Krishnan 1995).

**Table 2:** Rat physiological parameters and tissue/blood partition coefficients for PBPK modeling

Tissues	Blood flow^a^ (% CO)	Tissue volume^b^ (% BW)	Tissue/blood partition coefficients^c^		
			EGCg	ECg	EC
Adipose	7.00	7.60	0.20	0.75	0.08
Blood		8.16			
Bone	12.20	4.15	1.62	4.00	0.39
Brain	2.00	0.57	3.15	7.84	0.73
GI tract	13.10	2.70	2.04	4.82	0.63
Heart	4.90	0.33	1.27	2.65	0.62
Kidney	14.10	0.73	1.43	3.10	0.63
Liver	17.50	3.66	1.50	3.37	0.59
Lung		0.50	1.70	3.83	0.65
Muscle	27.80	40.40	1.02	1.97	0.59
Skin	5.80	19.00	1.79	4.23	0.55
Spleen	2.00	0.20	0.98	1.85	0.63
Rest of body	8.70	12.00	1.00	1.00	1.00

a CO (L/h), the cardiac output of rats, is scaled from the allometric equation, CO = 14.0 × (BW)^0.75^ (Travis 1987) The CO of a 0.4 kg rat is 7.08 L/h. Mean tissue blood flows are adapted from Luttringer et al. (2003) and Davies and Morris (1993).

b BW is the body weight of rats in kg. Mean tissue volumes are taken from Luttringer et al. (2003) and Davies and Morris (1993). Gut lumen volume is assumed to be 0.0176 L (Angelo and Pritchard 1987).

c Tissue/blood partition coefficients are estimated using the tissue composition model (Poulin et al. 2001; Poulin and Theil 2002).

**Table 3:** Pharmacokinetic parameters used to simulate the kinetics of individual tea catechins in rats after consuming a single dose of pure EGCg or PE

Parameters^a^	EGCg	ECg	EC
*k*_ac_ (/min·kg)^b^	0.003	0.002	0.002
*F*	0.0003–0.038	0.06	0.13
*tlag* (min)	10.00	10.00	5.00
*R*_t_ (min)	3.00	0.30	2.00
*k*_rac_ (/min·kg)^b^	0.67	0.41	13.40
*k*_fc_ (/min·kg)^b^	0.13	0.13	0.13
CL_bc_ (mL/min·kg)^c^	9.13	12.60	8.70
CL_rc_ (mL/min·kg)^d^	0.36	0.30	4.50

a The parameters are defined in the text.

b Rate constants are scaled from the body weight based on the following equations: *k*_a _= *k*_ac_(BW)^−0.3^, *k*_ra _= *k*_rac_(BW)^−0.3^, and *k*_f  _= *k*_fc_(BW)^−0.3^.

c CL_bc_ are the scaling coefficients of biliary clearance, CL_b_ which is scaled from body weight using the allometric equation, CL_b _= CL_bc_(BW)^0.66^.

d CL_rc_ are the scaling coefficients of renal clearance, CL_r_ which are derived experimentally from rats after receiving an *i.v*. dose of DGT (Zhu et al. 2001) and scaled to the body weight of rats in the present study using the allometric equation, CL_r _= CL_rc_(BW)^0.66^. The CL_r_ of EGC is assumed equal to EC.

#### Model structure

**Figure 2:**
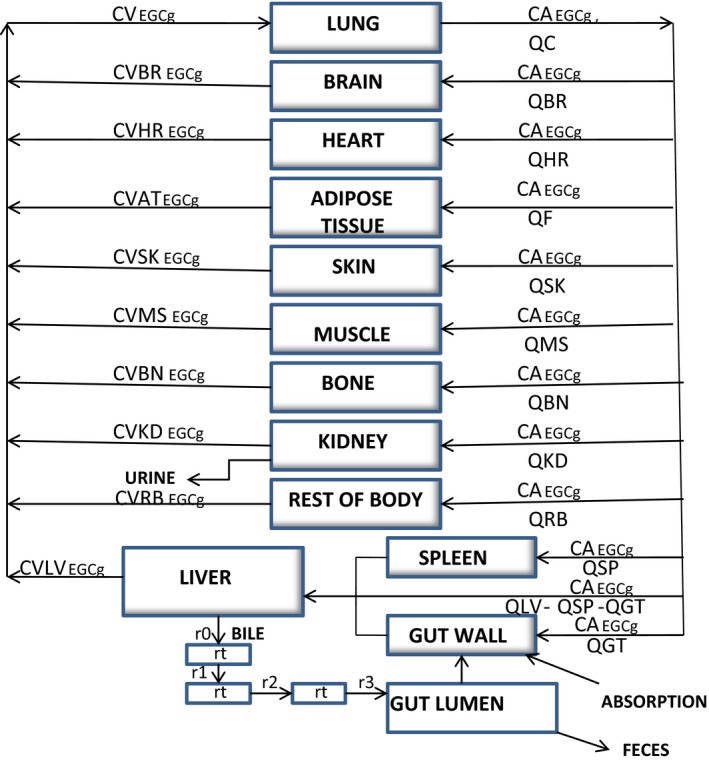
Schematic for the physiologically based pharmacokinetic description of a tea catechin in rats and humans. CA, CV, and Q, respectively, represent arterial concentrations, venous concentrations, and blood flows. The symbols and pharmacokinetic parameters are defined in the Appendix, and Tables 2, 4. The entero‐hepatic recycling sub‐model was adapted from Harrison and Gibaldi (1977) with modification.

Figure 2 shows a schematic for the PBPK model of a single tea catechin in rats or humans: (A) the model consisted of 13 first‐ordered, blood flow‐limited compartments including the lung, kidney, muscle, brain, liver, spleen, gut, bone, skin, heart, fat, blood, and rest of the body. Some of these tissue compartments were identified as the target organs of GT chemoprevention in rats (Yang et al. 2002), (B) individual TCs were absorbed into the blood *via* the gastrointestinal tract with absorption rate constants (*k*_a_). A lag time (*tlag*) was used to account for the time delay of absorption, (C) TCs were metabolized by the liver mainly to glucuronic acid/sulfate conjugates (Cai et al. 2002) before being excreted into the bile. These conjugated metabolites might be de‐conjugated by microorganisms in the colon and underwent entero‐hepatic recycling. The entero‐hepatic recycling model was modified from Bischoff et al. (1971) and Harrison and Gibaldi (1977). Biliary clearance (CL_b_) represented both the metabolic and secretory processes of the liver. The residence time (*R*_t_) represented the average time these metabolites spent in the bile before being reabsorbed (see Appendix A5). A reabsorption rate constant (*k*_ra_) was used to describe the reabsorption of TCs at a location different from the initial absorption sites.

#### Parameterization of PBPK model for rats

##### Physiological parameters

Tissue volumes and blood flows of rats with an average body weight (BW) of 0.26 kg were taken from the literature (Davies and Morris 1993; Luttringer et al. 2003). Tissue volumes were expressed as the percentage of average BW while blood flows were expressed as the percentage of cardiac output (CO) (Table 1). Gut content was assumed to be 0.014 mL for rats with an average BW of 0.25 kg (Angelo and Pritchard 1987). Physiological parameter values were scaled to the average BW of rats used in the pharmacokinetic study.

##### Tissue/blood partition coefficients

The tissue/blood PCs of EGCg, ECg, and EC (Table 2) were predicted using the tissue composition‐based equations (Poulin and Krishnan 1995; Poulin et al. 2001; Poulin and Theil 2002):

(1) $$\begin{array}{cc} P_{t:b\;\;\mathrm{non}-\mathrm{adipose}}=\; & [P_{o:w}\left(V_{\mathrm{nt}}+0.3V_{\mathrm{pht}}\right)+\left(V_{\mathrm{wt}}+0.7V_{\mathrm{pht}}\right)]/\\ & \left[P_{o:w}\left(V_{\mathrm{np}}+0.3V_{\mathrm{php}}\right)+\left(V_{\mathrm{wp}}+0.7V_{\mathrm{php}}\right)\right]\\ & \times [f_{\mathrm{up}}/\left(f_{\mathrm{ut}}\times \text{BLPLR}\right)] \end{array}$$

(2) $$\begin{array}{cc} P_{t:b\;\;\mathrm{adipose}}=\; & [D_{\mathrm{vo}:w}\left(V_{\mathrm{nt}}+0.3V_{\mathrm{pht}}\right)+\left(V_{\mathrm{wt}}+0.7V_{\mathrm{pht}}\right)]/\\ & [D_{\mathrm{vo}:w}\left(V_{\mathrm{np}}+0.3V_{\mathrm{php}}\right)+\left(V_{\mathrm{wp}}+0.7V_{\mathrm{php}}\right)]\times \\ & \left[f_{\mathrm{up}}/\text{BLPLR})\right] \end{array}$$

where *P*_t:b non‐adipose_ represents non‐adipose tissue/blood PCs, *P*_t:b adipose_ represents adipose tissue/blood PCs, *P*_o:w_ represents *n*‐octanol:water PCs of non‐ionized catechin, *D*_vo:w_ represents vegetable oil:water distribution for both the non‐ionized and ionized TCs at pH 7.4, *V*_wt,_ *V*_nt,_ and *V*_pht_ are the fractional weight of water, neutral fat, and phospholipids in the tissue, respectively, and *V*_wp,_ *V*_np,_ and *V*_php_ are the corresponding values in the plasma. *V*_wt,_ *V*_nt,_ *V*_pht_, *V*_wp,_ *V*_np,_ and *V*_php_ were taken from Poulin and Theil (2002). *D*_vo:w_ was derived from *P*_o:w_ and pK_a_ according to Poulin and Theil (2002).

The parameters used to implement the tissue composition‐based model (Table 2) were obtained as follows: (1) Log *P*_o:w_ and pK_a_ for individual TCs were predicted using the ACD/I‐Labs online engine (Advanced Chemistry Development Inc., Toronto, Ontario, Canada. https://ilab.acdlabs.com/). The predicted pK_a_ values were found to be very close to the experimental pK_a_ values which were 7.6, 7.6, >8.5 and >8.7 for EGCg, ECg, EGC, and EC, respectively (Inoue et al. 2002). (2) The BLPLR was calculated based on the theoretical partitioning of individual TCs into the erythrocytes and plasma of rats (Poulin and Krishnan 1995). Thus, BLPLR = (0.37 × P_e_  + 0.63 × *P*_p_)/*P*_p_, where *P*_e_ was the partitioning of the tea catechin into the erythrocytes of rats and *P*_p_ was the partitioning of the tea catechin in the plasma. Model‐predicted BLPLR were found to be very close to the experimental values which were 0.67, 0.61, and 0.87 for EGCg, ECg and EC, respectively (Zhu et al. 2001). (3) *F*_up_ and *f*_ut_ represent the unbound fractions of individual TCs in the plasma and tissue, respectively. The *f*_up_ of EGCg, ECg, and EC in the plasma of rats were determined experimentally to be 4%, 3% and 25%, respectively (Zhu et al. 2001). The *f*_up_ of EGC was not available; it was assumed equal to the EC value. The f_ut_ was calculated using the equation, *f*_ut _= 1/[1 +  (((1−*f*_up_)/*f*_up_) × 0.5)] (Poulin and Theil 2002).

##### Pharmacokinetic parameters

The *k*_a_ of EGCg and EC in rats was taken from Chen et al. (1997) study. The *k*_a_ of ECg was not available; it was assumed equal to that of EC (Table 3). The absorption rate constant scaling coefficients (*k*_ac_) were derived from the allometric equation, *k*_ac _= *k*_a_/(BW)^−0.3^ (Travis 1987). Renal clearance (CL_r_) of EGCG, ECg and EC were 0.15, 0.11, and 1.66 mL/min; (Zhu et al. 2000, 2001). The scaling coefficients (Cl_rc_) were derived from CL_r_ using the allometric equation, CL_rc _= CL_r_/(BW)^0.66^ (Chiou et al. 1998) (Table 3). Because TCs were metabolized by the liver and excreted into the bile, biliary clearance (CL_b_) was assumed equal to hepatic clearance (CL_h_) which was shown to be 3.74, 3.96, and 5.72 mL/min for EGCG, ECg, and EC, respectively (Zhu et al. 2001). Biliary clearance scaling coefficients (CL_bc_) were derived using the allometric equation, CL_bc _= CL_b_/(BW)^0.66^ (Chiou et al. 1998). Fecal transport rate constant (*k*_f_) (Lutz et al. 1977) was assumed to be 1/transit time in the small intestine as reported by Davies and Morris (1993). Final adjustments were made on the model parameters by fitting the PBPK model to the experimental data of Zhu et al. (2000). No further adjustment was allowed once the parameter values were finalized in Table 3.

### Scaling up of rat PBPK models to humans and parameterization of human models

The PBPK model of EGCg in rats (Fig. 2) was scaled up to humans by substituting the physiological parameters, pharmacokinetic parameters such as clearances, and PCs of the rat model with human specific values (Table 4). The human EGCg model was converted to an EGC or EC model by replacing the physicochemical parameters, pharmacokinetic parameters, and PCs with values specific for the tea catechin (Tables 4, 5).

**Table 4:** Human physiological parameters and tissue/blood partition coefficients for PBPK modeling

Tissues	Blood flow^a^ (% CO)	Tissue volume^b^(% BW)	Tissue/blood partition coefficients^c^		
			EGCg	EGC	EC
Adipose	5.00	12.00	0.15	0.01	0.01
Blood		7.71			
Bone	5.00	8.56	3.22	0.48	0.41
Brain	12.00	0.02	3.12	0.69	0.62
GI tract	17.00	1.71	2.49	0.53	0.55
Heart	4.00	0.47	1.00	0.51	0.50
Kidney	19.00	0.44	1.38	0.56	0.53
Liver	25.00	2.57	2.05	0.59	0.55
Lung		0.76	0.57	0.51	0.51
Muscle	17.00	40.00	1.38	0.54	0.52
Skin	5.00	3.71	1.60	0.53	0.50
Spleen	2.00	0.26	1.40	0.56	0.54
Rest of body	8.00	21.60	1.00	1.00	1.00

a CO (L/h) represents the cardiac output of human volunteers; it is scaled from the allometric equation, 16.1 (BW)^0.75^ (Travis 1987) based on the CO of 390 L/h for a 70 kg human. Mean data on blood flows are adapted from Luttringer et al. (2003).

b BW is the average body weight of human volunteers in kg. Mean data on tissue volumes are taken from Luttringer et al. (2003). Gut lumen volume is assumed to be 2.1 L (Bischoff et al. 1971).

c Tissue/blood partition coefficients are estimated using the tissue composition model (Poulin et al. 2001; Poulin and Theil 2002).

**Table 5:** Pharmacokinetic parameters used to simulate the kinetics of individual tea catechins in humans after consuming a single dose of pure EGCg or PE

Parameters^a^	EGCg	EGC	EC
*k*_ac_ (/h·kg)^b^	{1.1}[0.85](2.85)	2.19	1.86
*F*	{0.065}[0.12](0.07)	[0.013](0.052)	[0.01] (0.1)
*tlag* (h)	0.50	0.40	0.40
*k*_fc_ (/h·kg)^b^	25.60	25.60	25.60
*R*_t_ (h)	0.03	0.03	0.03
*k*_rac_ (/h ·kg)^b^	0.18	0.18	0.18
CL_bc_ (L/h ·kg)^c^	2.70	0.97	1.03
CL_rc_ (L/h ·kg)^d^	0.0023	0.34	0.56

a The parameters are defined in the text.

b Rate constants, *k*_a_, *k*_ra_, and *k*_f_ are up‐scaled from rats (see Table 4) using the following allometric equations: *k*_a _= *k*_ac_ (BW)^−0.3^, *k*_ra _= *k*_rac_ (BW)^−0.3^, and *k*_f_ = *k*_fc_ (BW)^−0.3^. Values in {}, [] and () brackets are used to simulate Chow et al. (2003), Chow et al. (2001) and Lee et al. (2002) studies, respectively.

c CL_bc_ is the scaling coefficient of biliary clearance, CL_b_ is up‐scaled from rats (see Table 3) using the allometric equation, CL_b _= CL_bc_(BW)^0.66^.

d CL_rc_ is the scaling coefficient of renal clearance. CL_r_ is calculated using the equation, CL_r _= amount of catechin in urine/AUTC_plasma_. Urinary excretion data are obtained from the studies of Meng et al. (2002) and Lee et al. (2002).

The human PBPK model of a single tea catechin was parameterized as follows: (1) human tissue volumes and blood flows to the tissues (Table 4) were taken from the literature (Luttringer et al. 2003). Tissue/blood PCs for humans (Table 4) were predicted using the tissue composition‐based model (Poulin et al. 2001; Poulin and Theil 2002). Gut content was assumed to be 2.1 L (Bischoff et al. 1971), (2) the CL_r_ of a tea catechin (Table 5) was estimated by dividing the amount excreted in the urine with the area under the curve in plasma (AUC_plasma_). Thus, the CL_r_ of EGCg was derived from the study of Meng et al. (2002) and the CL_r_ of EGC and EC were calculated from the study of Lee et al. (2002). The CL_rc_ of individual TCs were derived from their CL_r_ using the allometric equation, CL_rc _= CL_r_/(BW)^0.66^ (Chiou et al. 1998), (3) the CL_bc_ of individual TCs (Table 5) were scaled up from CL_b_ of rats using allometric equation, CL_bc _= CL_b_/(BW)^0.66^ (Chiou et al. 1998), and (4) human *k*_a_, *k*_ra_, and *k*_f_ rate constants (Table 5) were scaled up from rats using the equation, *k*_c _= *k*/(BW)^−0.3^, where *k* represented the rate constant, *k*_c_ represented the scaling coefficient, and BW was the average body weight of human volunteers (Travis 1987). Human BLPLR were assumed equal to those of rats (Table 1). Model parameters that could not be parameterized a prior were optimized by fitting the PBPK model to available experimental data (see Data fitting below). Final physiological and biochemical parameter values were summarized in Tables 4, 5, respectively.

### Construction of a PBPK model of tea catechin mixture for rats and humans

**Figure 3:**
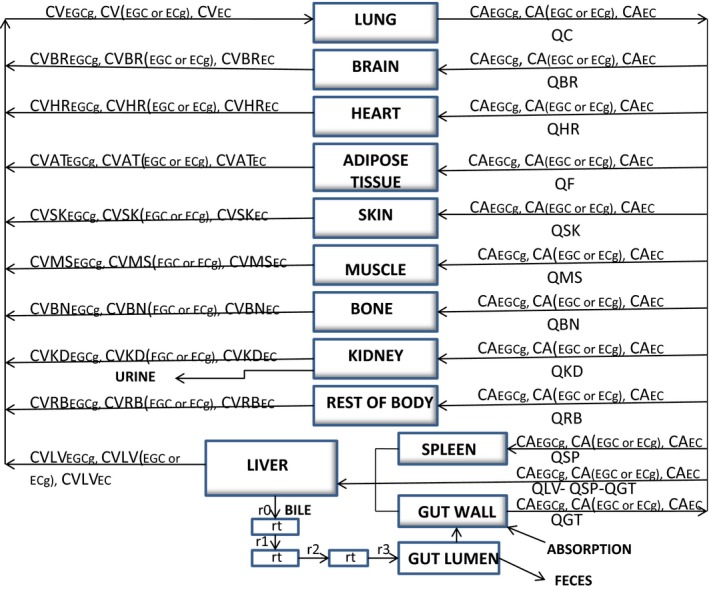
Schematic for the physiologically based pharmacokinetic description of a mixture of tea catechins in rats and humans. CA, CV, and Q, respectively, represent arterial concentrations, venous concentrations and blood flows. The symbols and pharmacokinetic parameters are defined in the Appendix, and Tables 2, 4. The entero‐hepatic recycling sub‐model was adapted from Harrison and Gibaldi (1977) with modification. For the sake of clarity, only the parameters of a single TC was shown in the enterohepatic recycling sub‐model.

A PBPK model of TCM was constructed by linking three different catechin models of rats (or humans) together without accounting for pharmacokinetic interactions between the TCs (Fig. 3). Pharmacokinetic interactions were assumed to be negligible or insignificant among the TCs or between a tea catechin and some unknown chemicals in GT or PE. The assumption of mixture PBPK model was tested by comparing model simulation with the PKs of three catechin constituents in the plasma of rats (or humans) after GT/PE consumption (see Results).

### Computer simulation and data fitting

#### Computer simulation

The differential and algebraic equations describing the movement of a single tea catechin (Fig. 2) or a mixture of TCs (Fig. 3) through the body of rats or humans were formulated as a computer program (see Appendix). After incorporating the different parameter values (Tables 1, 2, 3, 4, 5) into the PBPK model, the equations were solved numerically with the aid of AcslXtreme 2.5.0.6 (AEgis Technologies Group, Inc., Orlando, FL).

#### Data fitting

Model parameters that could not be parameterized a priori were estimated by fitting the initial estimates to the PBPK model with all other parameters fixed at the values listed in Tables 2, 3, 4, 5. Parameter values were adjusted manually at the beginning to test their effects on model simulation. Final adjustment of parameter values to fit the empirical data was carried out using the maximized log likelihood function of AcslXtreme OptStat (AEgis Technologies Group, Inc., Orlando, FL). Upper and lower bound limits on parameter values might be employed during the optimization process. Also, we might sacrifice the best‐fit to a dataset in order to obtain a set of parameter values which described the PKs of all other studies. Final parameter values that are adjusted to available experimental data are listed in Tables 1, 2, 3, 4, 5.

### Plasma dosimetry of tea catechin mixture

The *C*_max_ of individual TCs in a TCM were used as the dose surrogate (Andersen 1987; Ito et al. 2004) to calculate the integrated concentration/dose of TCM in the plasma. Thus, the mixture PBPK model for humans (Fig.  3) was used to predict the *C*_max_ of individual tea catechin constituents according to the experimental conditions described in Chow et al. (2001, 2005) and Lee et al. (2002) studies. The predicted *C*_max_ was multiplied by the inhibitory equivalence factor of the tea catechin in inhibiting hepatic 7‐ethoxyresorufin‐*O*‐deethylase (EROD) activity in vitro (Obermeier et al. 1995). The products of multiplication were added together yielding the integrated concentration/dose of TCM in the plasma (*μ*g EGCg equivalents/mL) of humans after GT/PE consumption. Similarly, the TCM dose metric in externally administered PE/GT was calculated but expressed as *μ*g EGCg equivalents/g of GT/PE. The different steps involved in calculating the dose metrics of TCM were summarized in the following concentration/dose additivity equation (ATSDR, 2004; EPA 2007):

(3) $$\begin{array}{cc} \text{TCM concentration/dose}=\; & C_{1}\times {\text{IEF}}_{1}+C_{2}\times {\text{IEF}}_{2}\\ & +\ldots +C_{n}\times {\text{IEF}}_{n}\\ & =\sum_{i=1}^{n}Ci\times {\text{IEF}}_{i} \end{array}$$

where, TCM concentration/dose is expressed in *μ*g EGCg equivalents/g or mL, *C*_1_ represents the concentration of EGCg in PE or the *C*_max_ of EGCg in the plasma, C_*i*_ represents the *i*th tea catechin in a TCM, IEF_1_ represents the inhibitory equivalence factor of EGCg which is assigned a value of 1.00, and IEF_*i*_ is the inhibitory equivalence factor of the *i*th tea catechin constituent relative to EGCg. The IEF of EGCg, EGC, ECg, and EC are 1.00, 0.85, 0.45, and 2.21, respectively, since the EROD IC_50_ are 1175, 1000, 530, and 2600 *μ*mol/L, respectively, in human liver microsomes (Obermeier et al. 1995).

The predicted TCM dose metrics in the plasma of humans from different pharmacokinetic studies were plotted against the administered doses. The plot was subjected to linear regression analysis using the GraphPad Prism Software version 5.04 (San Diego, CA).

### Statistical and sensitivity analyses

#### Mean absolute prediction error

Mean absolute prediction error (MAPE) was used as a measure of good fit between model‐predicted concentration (*C*_predi_) and experimental concentration (*C*_expti_). It was calculated using the following equation:

(4) $$\mathrm{MAPE}(\%)=\left(100/N\right)\left[(\sum_{i=1}^{n}\times |C_{\mathrm{expti}}-C_{\mathrm{predi}}|/C_{\mathrm{expti}})\right]$$

where, *i* represents individual data points and N is the total number of data points. A deviation within a factor of two between predicted and experimental concentration data (i.e., MAPE<50%) was used as the criteria for goodness of fit (Bjorkman et al. 1994).

#### Log‐normalized sensitivity parameter

Log‐normalized sensitivity parameter (LSP) was used to identify key model parameters that had significant impacts on model prediction. LSP is defined by the following equation (Clewell et al. 1994):

(5) LSP=δlnR/δlnX

where R is the model output and X is the parameter for which the sensitivity is assessed. This equation quantified the percentage change in an output value as a result of the percentage change in a parameter. The sensitivity analysis was conducted using AcslXtreme 2.5.0.6 (AEgis Technologies Group, Inc., Orlando, FL). The sensitivities of EGCg plasma concentration to tissue/blood partition coefficients and pharmacokinetic parameters were determined in rats (Zhu et al. 2000) and humans (Chow et al. 2001) after consuming a dose of pure or crude EGCg. The LSP of parameters were determined at the last time point of the study using the central difference method (Clewell et al. 1994). A LSP > 1 indicated errors in the input parameter resulted in amplified errors in the model output, which was an undesirable feature of the model.

## Results

### Development of a PBPK model of EGCg for rats and humans

**Figure 4:**
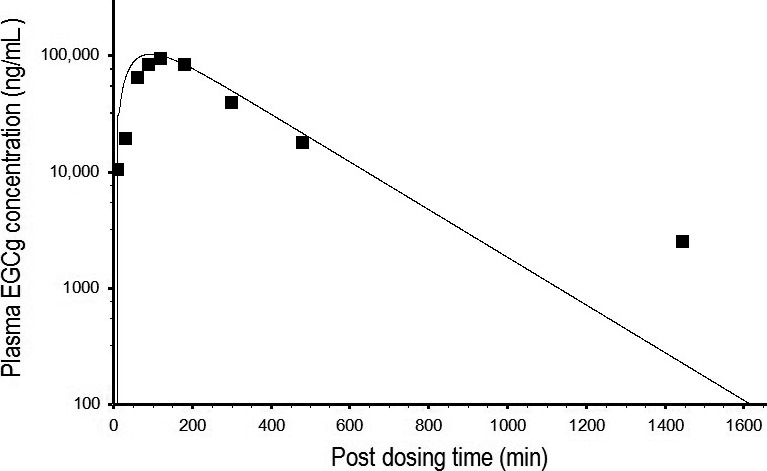
Predicted and measured time course of free EGCg concentrations in the plasma of rats after consuming a single dose of 2500 mg/kg crude EGCg from a PE preparation. ▄ represents mean concentrations of free EGCg (*N* = 6) at different time points post‐dosing (Zhu et al. 2000). _________ represents model‐simulated concentration‐time curve of free EGCg.

**Figure 5:**
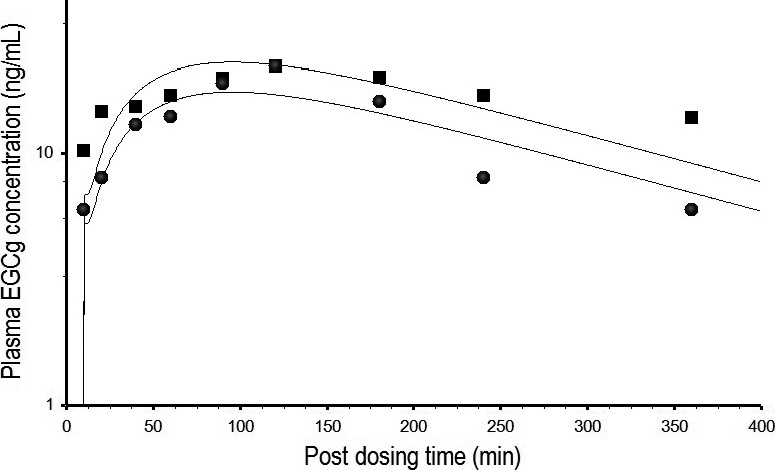
Predicted and measured EGCg concentrations in the plasma of rats after consuming a single dose of pure EGCg (75 mg/kg) or crude EGCg (14.6 mg/kg) from a PE formulation. ▄ and ● represent the time course of total EGCg (free EGCg plus conjugated forms) concentrations in the plasma of rats after consuming pure EGCg and crude EGCG, respectively (Chen et al. 1997). __________ represents model‐simulated concentration‐time curve of free EGCg in the plasma of rats after consuming pure EGCg (upper curve) or crude EGCg (lower curve).

A PBPK model of EGCg was developed by comparing model simulation with free EGCg concentration‐time data in the plasma of rats after consuming 2500 mg/kg of crude EGCg in PE (Zhu et al. 2000). Figure 4 shows the time course of predicted and observed EGCg concentrations in the plasma.The goodness of fit between predicted and measured data was judged by the 34.9% MAPE which showed predicted EGCg concentrations were within the two‐fold error range of empirical EGCg concentrations. The EGCg model of rats was validated by another set of kinetic data from rats after receiving an oral dose of pure EGCg (75 mg/kg) or crude EGCg (6 mg/kg) in PE (Chen et al. 1997). The EGCg model of rats also was able to duplicate the PKs of pure EGCg and crude EGCg with 17.4% and 14.6% MAPE, respectively (Fig. 5). Simulated concentration‐time curves for pure EGCg appeared parallel to those of crude EGCg. If predicted EGCg concentrations in the plasma were normalized by the administered dose (75 mg/kg for pure EGCg and 6 mg/kg for crude EGCg) before plotting against sampling times, the concentration‐time curve of pure EGCg would be superposable on that of crude EGCg. These results indicated that the concentration‐time curves of pure EGCg and crude EGCg in Figure 5 were indeed parallel to each other (Gabrielsson and Weiner 2000). In other words, the PKs of pure EGCg and crude EGCg were very similar in rats.

**Figure 6:**
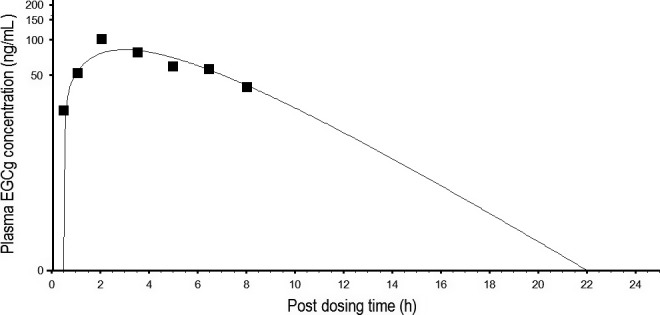
Predicted and measured free EGCg concentrations in the plasma of humans after consuming a single dose of 400 mg pure EGCg. ▄ represents mean concentrations of free EGCg (*N* = 8) at different time points post‐dosing (Chow et al. 2003). __________ represents model‐simulated concentration‐time curve for free EGCg.

**Figure 7:**
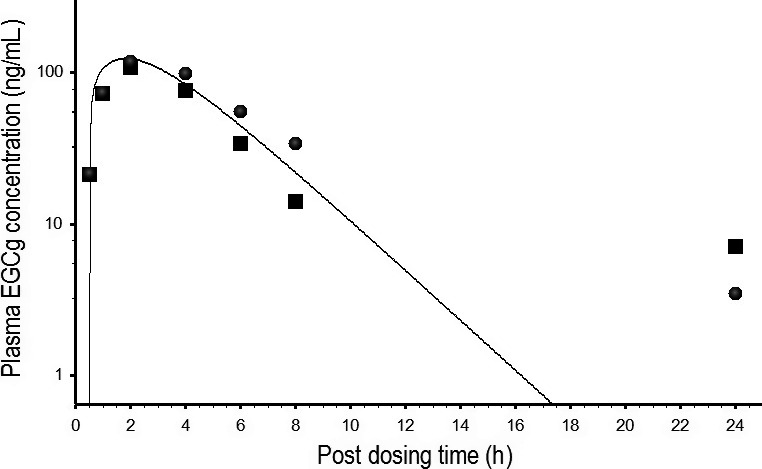
Predicted and measured free EGCg concentrations in the plasma of humans after consuming 400 mg pure EGCg or 400 mg crude EGCg from a PE formulation. ▄ and ● represent mean concentrations of free EGCg (N = 5) in the plasma of humans after consuming pure EGCg and crude EGCg, respectively (Chow et al. 2001). ___________ represents the simulated concentration‐time curves of humans after consuming pure EGCg or crude EGCg; these curves are identical because both EGCg formulations contain the same amount (400 mg) of EGCg (Chow et al. 2001),

The rat EGCg model was scaled up to humans by replacing the physiological parameters, PCs and pharmacokinetic parameters with human‐specific values (Tables 4, 5). The human EGCg model was calibrated by observed data from Chow et al. (2003) study. Figure 6 shows the predicted and actual kinetic profiles of EGCg in the plasma of humans. The human EGCG model was able to duplicate the observed data since the MAPE between predicted and actual data was only 13.2%. The human EGCg model was validated by another set of kinetic data from humans after consuming 5.33 mg/kg of pure EGCg or 5.33 mg/kg crude EGCg in PE (Chow et al. 2001). The human EGCg model also was able to reproduce the observed data of pure EGCg and crude EGCg with 25.9% and 30.8% MAPE, respectively, although EGCg concentrations were under‐predicted at or close to the 24‐h, post‐dosing time point (Fig. 7). The predicted concentration‐time profiles for pure EGCg and crude EGCg were identical (Fig. 7) because both EGCg formulations contained the same dose (5.33 mg/kg) of EGCg (Chow et al. 2001).

### Sensitivity analysis of EGCg PBPK model

Table 6 shows the LSP of pharmacokinetic parameters and tissue/blood PCs with respect to EGCg concentrations in the plasma of rats and humans. The LSP of F, BLPLR, and k_ac_ in human EGCg model were all >1.0 in absolute values. By contrast, the LSP of tissue/blood PCs, except those of Rms and Rrb, were all <1.0 (Table 6). Similarly, the LSP of F, BLPLR, and BW were >1.0 and the LSP of tissue/blood PCs were <1.0 in rat EGCg model (Table 6).

**Table 6:** Log‐normal sensitivity analysis ‐ the effects of pharmacokinetic parameters and partitioning coefficients on plasma EGCg concentration‐time profile

Pharmacokinetic parameters^a^	Sensitivity coefficients		Partitioning coefficients^a^	Sensitivity coefficients	
	Rat^b^	Human^c^		Rat^b^	Human^c^
BW	10.1	—	Rbn	—	—
BLPLR	–1.1	–1.1	Rbr	—	—
CL_bc_	—d	—	Rft	—	—
CL_rc_	—	–0.7	Rgt	—	—
*F*	29.4	15.2	Rhr	—	—
*R*_t_	—	—	Rkd	—	—
*k*_ac_	—	−4.8	Rlg	—	—
*k*_fc_	—	—	Rlv	—	—
*k*_rac_	—	—	Rms	—	1.9
*tlag*	—	0.8	Rrb	—	1.2
			Rsk	—	—
			Rsp	—	—

a See Tables 2, 3, 4, 5 and Appendix for explanation on parameter abbreviations.

b Rats are given a single oral dose of PE containing 2500 mg/kg crude EGCg (Zhu et al. 2000).

c Humans are given a single oral dose of pure EGCg (5.33 mg/kg) (Chow et al. 2001).

d – Represents less than 0.5 in absolute value.

### Construction of a PBPK model of tea catechin mixture in rats and humans

**Figure 8:**
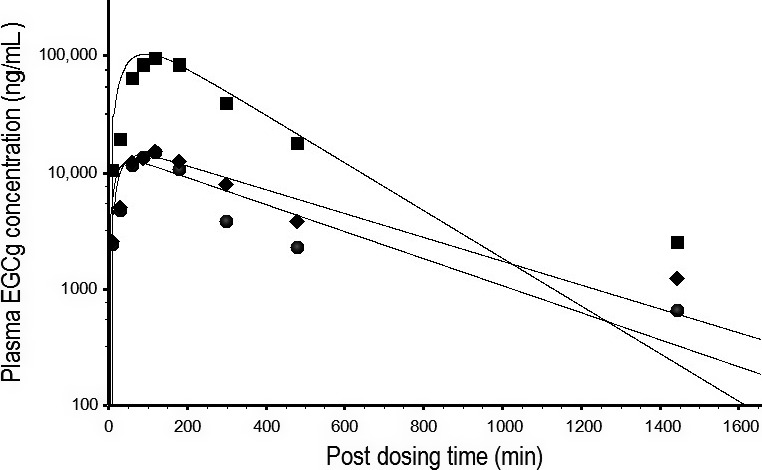
Predicted and measured free EGCg, ECg, and EC concentrations in the plasma of rats after consuming PE containing a mixture of EGCg (2500 mg/kg), ECg (650 mg/kg), and EC (250 mg/kg). ▄, ♦, and ●, respectively, represent mean concentrations (*N* = 6) of free EGCg, ECg, and EC at different time points post‐dosing (Zhu et al. 2000). ___________ represents model‐simulated concentration‐time curves for free EGCg (top), ECg, (middle), and EC (bottom). Note: the EGCg concentration‐time curve is taken directly from Figure 4 for comparison.

The rat EGCg model was extrapolated to an ECg or EC model by replacing the physicochemical parameters, pharmacokinetic parameters, and PCs with ECg‐ or EC‐specific values (Tables 1, 2, 3). A PBPK model of TCM for rats (Fig. 3) was constructed by linking the EGCg, ECg, and EC models together under the assumption of no pharmacokinetic interaction between the TCs. The PBPK model of TCM was calibrated by the kinetic data of rats after receiving an oral dose of PE containing a mixture of EGCg (2500 mg/kg), ECg (650 mg/kg), and EC (250 mg/kg) (Fig. 2 of Zhu et al. 2000). Figure 8 shows the predicted and observed kinetic profiles of EGCg, ECG, and EC in the plasma of rats. The PBPK model of TCM was able to describe the observed data closely since the overall MAPE between predicted and measured data was only 33.9%.

**Figure 9:**
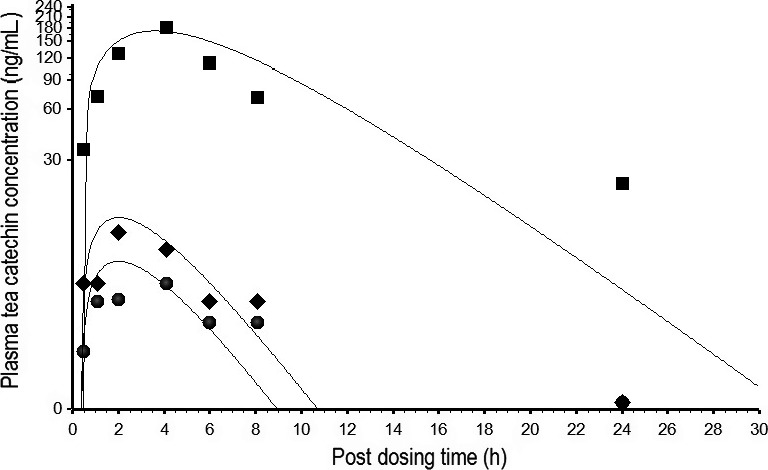
Predicted and measured free EGCg, EGC, and EC concentrations in the plasma of humans after consuming PE containing a mixture of EGCg (8.3 mg/kg), EGC (1.54 mg/kg), and EC (1.29 mg/kg). ▄, ♦, and ●, respectively, represent mean plasma concentrations (*N* = 5) of EGCg, EGC, and EC at different time points post‐dosing (Chow et al. 2001); __________ represents model‐simulated concentration‐time curves for free EGCg (top), EGC (middle), and EC (bottom).

**Figure 10:**
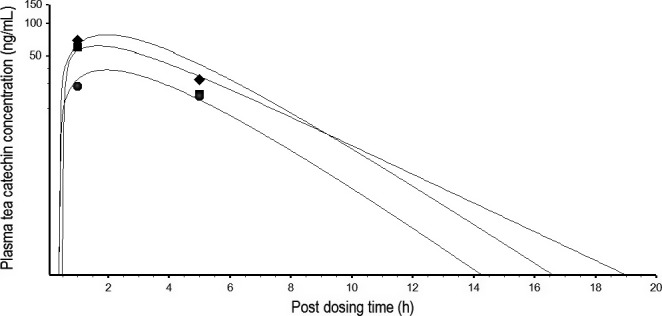
Predicted and measured free EGCg, EGC, and EC concentrations in the plasma of humans after consuming green tea solids containing a mixture of EGCg (2.78 mg/kg), EGC (2.20 mg/kg), and EC (0.64 mg/kg). ♦, ▄, and ●, respectively, represent mean plasma concentrations (*N* = 5) of EGCg, EGC, and EC at different time points post‐dosing (Lee et al. 2002). _______ represents model‐simulated concentration‐time curves for EGCg (top), EGC (middle), and EC (bottom).

The human EGCg model was extrapolated to an EGC or EC model by substituting the physicochemical parameters, pharmacokinetic parameters, and PCs with EGC‐ or EC‐specific values (Tables 1, 4 and 5). A PBPK model of TCM was constructed for humans (Fig. 3) by linking the EGCg, EGC, and EC models together with no pharmacokinetic interaction between the TCs. The PBPK model of TCM was calibrated with the kinetic data of humans after consuming PE containing a mixture of EGCg (8.3 mg/kg), EGC (1.54 mg/kg), and EC (1.29 mg/kg) (Chow et al. 2001). Figure 9 shows the predicted and observed kinetic profiles of EGCg, EGC, and EC in the plasma of humans. The TCM model was able to reproduce the observed data; the MAPE between predicted and measured concentrations was 29.6%. The TCM model was further validated by comparing model simulation with observed data from humans after consuming GT solids containing a mixture of EGCg (2.78 mg/kg), EGC (2.2 mg/kg), and EC (0.64 mg/kg) (Lee et al. 2002). Figure 10 shows the predicted and actual concentration‐time profiles of the tea catechin constituents in the plasma. The PBPK model was able to describe the observed data closely and the MAPE of the model was just 20.98%.

### Using model‐predicted C_max_ to calculate TCM dosimetry in the plasma of humans

**Figure 11:**
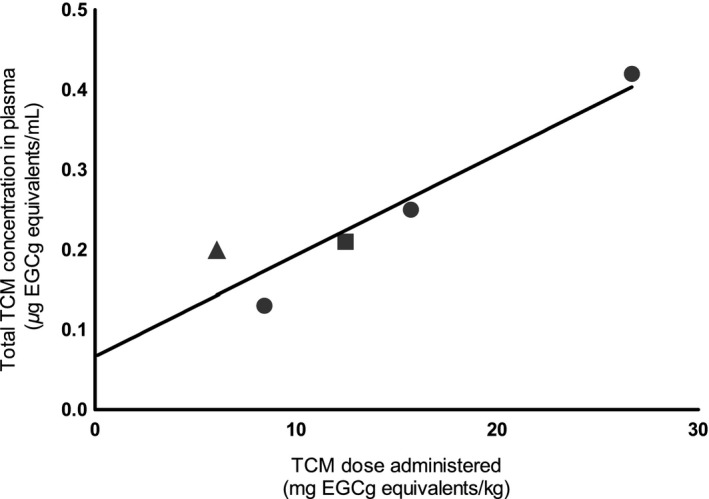
Quantitative relationship between total TCM concentration in plasma and applied dose in humans. TCM dose metrics, expressed in *μ*g EGCg equivalents/mL plasma or g of PE, are calculated using the concentration addition model of ATSDR (2004). ● represents total TCM concentrations in the plasma of humans after consuming 400, 800, or 1200 mg PE (Chow et al. 2005); ▲ and ▄ represent total TCM concentrations in the plasma of humans after consuming 20 mg/kg of green tea solids (Lee et al. 2002) and 600 mg of PE (Chow et al. 2001), respectively.

Table 7 (column 2) lists the predicted *C*_max_ (no bracket) and measured *C*_max_ (bracketed) of EGCg, EGC, and EC from different pharmacokinetic studies. Predicted C_max_ are very close to measured *C*_max_ except the *C*_max_ of EGC and EC in Lee et al. (2002) study and the *C*_max_ of EGCg (1200 mg PE) in Chow et al. (2005) study. The effect‐based or integrated TCM concentrations in the plasma of humans were calculated using the concentration addition approach (ATSDR 2004) which assumed individual tea catechin concentrations in the TCM were additive after adjusting for their inhibition potencies on hepatic EROD activities (Table 7, column 3). When the total TCM concentrations in the plasma of humans were plotted against the administered doses (Table 7, column 4), a straight line with a slope of 0.013 ± 0.003 (*R* ^2 ^= 0.88) was obtained (Fig. 11).

**Table 7:** Using *C*_max_ as a dose surrogate to predict plasma dosimetry of TCM in humans

GT solids and PE pharmacokinetic studies in humans	Predicted and actual *C*_max_ in the plasma (*μ*g/mL)			Total TCM concentration in the plasma (*μ*g EGCg equivalents/mL)^d^	Administered TCM dose metrics (mg EGCg equivalents/kg)^e^
	EGCG	EGC	EC		
Lee et al. (2002); 20 mg/kg GT solids	0.06^a^ (0.08)^b^	0.07 (0.22)^c^	0.04 (0.12)^c^	0.20	6.06
Chow et al. (2005); 400 mg PE	0.11 (0.14)	0.01 (0.02)	0.01 (0.00)	0.13	8.42
Chow et al. (2001); 600 mg PE	0.21 (0.17)	0.02 (0.01)	0.01 (0.01)	0.21	12.46
Chow et al. (2005); 800 mg PE	0.21 (0.29)	0.02 (0.03)	0.01 (0.00)	0.25	15.71
Chow et al. (2005); 1200 mg PE	0.35 (0.92)^c^	0.04 (0.04)	0.02 (0.01)	0.42	26.70

a Values without brackets are predicted *C*_max_ of unchanged TCs based on PBPK modeling.

b Bracketed values are *C*_max_ from the literature. These *C*_max_ usually are determined from free tea catechin concentrations except those of Lee et al. (2002) study, which are determined from total tea catechin concentrations (free plus conjugated forms).

c C_max_ is under‐predicted when compare with the observed value.

d Total TCM concentrations are expressed as *μ*g EGCg equivalents/mL plasma; they are calculated using predicted *C*_max_ and the concentration/dose additivity model (ATSDR, 2004).

e Administered dose metrics are expressed as mg EGCg equivalents/kg BW; they are calculated using the concentrations of individual TCs in PE and the additivity model (ATSDR, 2004).

## Discussion

The PBPK model of EGCg (Fig. 2) is able to reproduce the concentration‐time profiles of free EGCg (Fig. 4) as well as total EGCg (free EGCg plus conjugated forms) (Fig. 5) in the plasma of rats. This is unexpected since the PBPK model supposedly is predictive for the kinetics of free EGCg only. The finding that our model is also predictive for the kinetics of total EGCg indicates the presence of free EGCg mainly in the plasma of rats. Thus, although Chen et al. (1997) have analyzed rat plasma samples for total EGCg, they actually measure free EGCg in these samples. Our hypothesis is supported by the following experimental evidence: (1) only free EGCg is found in the plasma of humans after consuming pure EGCg or PE (Chow et al. 2001, 2003; Lee et al. 2002), and (2) little or no EGCg conjugated metabolites are present in the blood of rats because most EGCg is metabolized by bacteria in the intestine (Kohri et al. 2001a).

Model‐simulated pure EGCg and crude EGCg concentration‐time curves are parallel to each other indicating the PKs of these two forms of EGCg are very similar in rats (Fig. 5). In contrast, Chen et al. (1997) have shown that the PKs of pure EGCg and crude EGCg are different in the plasma of rats. The discrepancy in results between our and Chen et al. (1997) studies may be explainable by the different pharmacokinetic approaches used to fit or analyze the empirical data: Chen et al. (1997) have fitted total EGCg concentration‐time curves with data‐based, non‐compartmental analysis yielding inaccurate, composite pharmacokinetic parameter values (Gabrielsson and Weiner 2000). As a result, Chen et al. (1997) may err in concluding that the PKs of pure EGCg and crude EGCg are different in rats. In contrast, the EGCg model (Fig. 2) is developed based on the physiological parameters of rats, and the physicochemical and pharmacokinetic characteristics of EGCg. As such, the predicted concentration‐time profiles are not affected significantly by errors in the empirical data. Andersen (1987) has shown that the PBPK model is better than classical pharmacokinetic approaches in data interpretation. Our results are in agreement with their findings.

We have integrated more than 40 parameters from different sources (Tables 1, 2, 3, 4, 5) to implement the PBPK model of EGCg (see Appendix). The effect of a parameter on model prediction can be quantified using sensitivity coefficient analysis (Clewell et al. 1994). Table 6 shows the LSP of most pharmacokinetic parameters and tissue/blood PCs are <0.5 in absolute values. Thus, errors in estimating the parameters and PCs would not affect significantly the predicted EGCg PKs in rats or humans. In contrast, the LSP of BLPLR, F, *k*_ac_, Rms and Rrb in human EGCg model, and the LSP of BLPLR, BW and F in rat EGCg model are >1 in absolute values (Table 6). Thus, small errors in estimating these parameters would impact significantly the predicted plasma concentration‐time profiles of EGCg. Because very few model parameters and PCs show LSP >1, errors in estimating model parameter values would not be significantly amplified in the predicted concentration‐time profiles.

Individual TCs in the TCM are assumed not to interact metabolically with one another in the PBPK model (Fig. 3). This assumption is supported by the following lines of evidence: (1) the PKs of pure EGCg and crude EGCg are similar in the plasma of rats (Fig. 5) and humans (Fig. 7). These results clearly demonstrate crude EGCg does not interact significantly with other TCs or unknown chemicals in GT/PE; (2) the mixture PBPK model is able to reproduce the PKs of three TCs simultaneously in the plasma of rats and humans after GT/PE consumption (Figs. 8, 9, 10). The difference in predicted and actual concentrations is small and probably is related to using reference instead of actual physiological parameters in PBPK modeling (Tables 2, 4) and large variation in empirical data (Zhu et al. 2000; Chow et al. 2001). If there were significant interactions between the TCs, we would observe much larger deviations between model simulation and empirical data; (3) Hong et al. (2001) have reported that systemic tea catechin concentrations in humans are about 5–50 folds less than the effective concentrations of in vitro studies. Tea catechin levels in human plasma (Figs. 8, 9, 10) probably are below the in vivo interaction thresholds since TCs have low oral bioavailabilities (Chow et al. 2005); and (4) hepatic glucuronyltransferase and sulfatase activities are difficult to modulate with chemical treatments. Thus, most drugs or chemicals are weak inhibitors of glucuronyltransferases (Resetar et al. 1991). TCs also are unable to interact with one another by competing for the active sites on catechol‐*O*‐methyltransferase (Meng et al. 2002), and CYP1A2 enzymes (Obermeier et al. 1995) as these are minor pathways of TCs metabolism.

The mixture PBPK model often under‐predicts the concentrations of TCs in the plasma of rats and humans at or near the 24‐h, post‐dosing time point (Figs. 7, 8, 9). An explanation for under‐predicting the observed data is not available but may be related to the detection limits of the analytical methods in these studies. Since the PBPK model does not have any detection limit, it is capable of predicting TCs at levels much lower than HPLC analysis. Other possible but unlikely explanations for under‐predicting the observed data include the inhibition of efflux transporters in rats and humans since plasma EGCg concentration is increased in humans after daily treatment with a high dose of PE (800 mg) for 4 weeks (Chow et al. 2003). Also, flaws in model structure and inaccurate model parameter values may play important roles in under‐predicting the observed data at or near the 24‐h time point.

A single set of parameter values, except F and k_ac_, has been used successfully to simulate the PKs of a tea catechin in different pharmacokinetic studies (Tables 3, 5). *K*_ac_ represents the absorption rate constant of TCs while *F* is the empirical bioavailability factor of the model, which does not equate to the absolute bioavailability of classical pharmacokinetic model (Anderton et al. 2004). Different *F* and/or *k*_ac_ values (Tables 3 and 5) are used to simulate the PKs of a tea catechin in different studies because systemic availability of TCs is highly variable in rats (Zhu et al. 2000) and humans (Chow et al. 2001; Lee et al. 2002). *F* and *k*_ac_ variation may be related to the fasting/fed conditions of the experimental animals since Chow et al. (2005) have reported that EGCg concentration is higher in the plasma of humans under fasting condition. It is interesting to note that the PCs of gallated TCs (e.g., ECg and EGCg) are larger than non‐gallated TCs (e.g., EGC and EC) (Tables 2). These imply gallated TCs are more widely distributed to the target organs of rats and humans than non‐gallated TCs. Indeed, EGCg is more widely distributed to the target organs of rats than EGC or EC (Chen et al. 1997). Similarly, biliary clearance (CL_bc_) is larger than renal clearance (CL_rc_) (Tables 3) indicating TCs are excreted into the bile instead of the urine, a finding which is also reported by Kohri et al. (2001b) after injecting rats i.v. with EGCg.

The effect‐based or integrated concentration of TCM in the plasma of humans is calculated using the C_max_ of individual TCs as dose surrogates. Table 7 shows predicted *C*_max_ are very close to observed *C*_max_ except the *C*_max_ of EGC and EC in Lee et al. (2002) study and the *C*_max_ of EGCg (1200 mg PE) in Chow et al. (2005) study. An explanation for the different predicted and observed *C*_max_ values in the EGCg study (Chow et al. 2005) is not available. On the other hand, the *C*_max_ of EGC and EC are under‐predicted because free TCs are predicted by the model whereas both free TCs and their conjugated metabolites are present in the plasma of humans (Lee et al. 2002). Despite the difference in predicted and measured *C*_max_ (Lee et al. 2002; Chow et al. 2005), total TCM concentration in the plasma is linearly related to the administered dose (Fig. 11). These results also validate the use of first‐order kinetics for tea catechin modeling (Figs. 2, 3).

This is the first study in which a mixture PBPK model is used to predict the PKs and systemic dosimetry of a ternary TCM in humans. The mixture model may be modified to simulate more than three TCs by linking additional tea catechin model(s) to the global model as described in the study of Haddad et al. (1999). The TCM model is a powerful tool for species‐to‐species and dose‐to‐dose extrapolation of pharmacokinetic data (Angelo and Pritchard 1987; Travis 1987). It can be used to estimate an internal tissue concentration/dose of GT/PE for safety assessment and dose‐response analysis. It is also useful in reducing costs and time of a GT/PE clinical study by better planning and study design (IPCS, 2010). The modeling framework as described in this paper is also applicable to plant‐based traditional medicines, functional foods, and dietary supplements.

## Disclosure

None declared.

## Appendix

The algebraic and mass balance differential equations which describe the movement of a tea catechin in rats or humans are formulated as a computer program to predict the concentration‐time profile of a tea catechin in arterial blood (CBA_c_), venous blood (CBV_c_), lung (CLG_c_), heart (CHR_c_), kidney (CKD_c_), adipose tissue (CFT_c_), muscle (CMS_c_), brain (CBR_c_), liver (CLV_c_), spleen (CSP_c_), gut tissue (CGT_c_), skin (CSK_c_), bone (CBN_c_), gut contents (CGC_c_), and the rest of the body (CRB_c_) (Figs. 2 and 3).

## Non‐eliminating organ

Lung, brain, spleen, muscle, bone, skin, adipose tissue, heart, and the rest of body:

(A1) $$\text{VX}\left({\text{dC}}_{c}/\text{dt}\right)=\text{QX}\left({\text{CBA}}_{c}-C_{c}/{\text{RX}}_{c}\right)$$

where, dC_c_/dt is the rate of catechin concentration change in the non‐eliminating organ; X represents the organ; QX, VX, and RX_c_ represent tissue blood flow, volume, and tissue/blood partition coefficient of the organ, respectively; subscript c represents EGCg, EGC, ECg, or EC. For example,

Lung:

(A2) $$\text{VLG}\left({\text{dCLG}}_{c}/\text{dt}\right)=\text{QC}\left({\text{CBV}}_{c}-{\text{CLG}}_{c}/\text{RLG}\right)$$

## Eliminating organs

Kidney:

(A3) $$\text{VKD}\left({\text{dCKD}}_{c}/\text{dt}\right)=\text{QK}\left({\text{CBA}}_{c}\;-{\text{CKD}}_{c}/{\text{RKD}}_{c}\right)-{\text{CL}}_{\mathrm{rc}}\left({\text{CKD}}_{c}/{\text{RKD}}_{c}\right)$$

where, CL_rc_ represents the renal clearance for the tea catechin.

Liver:

(A4) $$\begin{array}{cc} \text{VLV}({\text{dCLV}}_{c}/\text{dt})= & \left(\text{QLV}-\mathrm{QGT}-\mathrm{QSP}\right){\text{CBA}}_{c}\\ & +\text{QGT}({\text{CGT}}_{c}/{\text{RGT}}_{c})\\ & +\text{QSP}({\text{CSP}}_{c}/{\text{RSP}}_{c})\\ & -\text{QLV}\left({\text{CLV}}_{c}/{\text{RLV}}_{c}\right)-{\text{RAM}}_{c} \end{array}$$

$${\text{RAM}}_{c}={\text{CL}}_{bc}\left({\text{CLV}}_{c}/{\text{RLV}}_{c}\right)$$

(A5) $$R_{tc}\left({\text{dR}}_{\mathrm{cj}}/\text{dt}\right)=R_{\mathrm{cj}-1}-R_{\mathrm{cj}}\;\;j=0,1,2,\ldots n$$

where, RAM_c_ represents the combined rate of catechin and glucuronide transport from the liver cells into the bile duct; CL_bc,_ the biliary clearance, represents a combination of metabolic and excretory processes of the catechin in the liver; R_*tc*_, the residence time (bile volume/bile flow rate) which is assumed to be equal in each bile duct sub‐compartments; R_0_ is the transport rate into the first sub‐compartment and $R_{0}={\text{RAM}}_{c}$; R_cj_ is the transport rate from the jth duct sub‐compartment and *n* = 3 is the optimal number of sub‐compartment for the proposed PBPK model. The mass balance equations describing the time lag of biliary excretion with a series of bile duct sub‐compartments were adapted from Bischoff et al. (1971) with modification.

Gut contents:

(A6) $$\text{VGC}\left({\text{dCGC}}_{c}/\text{dt}\right)=R_{c3}-k_{\mathrm{fc}}\left({\text{CGC}}_{c}\right)\left(\text{VGT}\right)-k_{\mathrm{rac}}\left(\text{VGC}\right)*{\text{CGC}}_{c}$$

Gut tissue:

(A7) $$\text{VGT}\left({\text{dCGT}}_{c}/\text{dt}\right)=\text{QGT}\left({\text{CBA}}_{c}-{\text{CGT}}_{c}/{\text{RGT}}_{c}\right)+k_{\mathrm{rac}}*\text{VGC}\left({\text{CGC}}_{c}\right)+{\text{RAO}}_{c}$$

$${\text{RAO}}_{c}=k_{\mathrm{ac}}\left(F_{c}\right)\left({\text{dose}}_{c}\right)\exp^{-\mathrm{kac}(t-\mathrm{tlagc})}\;\text{for}\;\;t>\text{tlagc}$$

where RAO_c_ is an input function describing the absorption of catechin after oral administration, *k*_ac_ is a first‐order absorption rate constant from the small intestine, dose represents either the dose of a pure catechin or individual tea catechins in a PE preparation, *F*_c_ is the apparent or empirical bioavailability factor, and *t*_lagc_ is the lag time for absorption, *k*_rac_ is the re‐absorption rate constant of the tea catechin from the colon which is assumed different to the absorption of tea catechin from the small intestine, and *k*_fc_ is the fecal transport rate constant.

## Blood compartment

The total blood volume was divided into a two‐thirds venous pool and one‐third arterial pool.

Arterial blood:

(A8) $$\mathrm{VBA}\left({\mathrm{dCBA}}_{c}/\mathrm{dt}\right)=\mathrm{QC}\left({\mathrm{CLG}}_{c}/{\mathrm{RLG}}_{c}-{\mathrm{CBA}}_{c}\right)$$

Venous blood:

(A9) $$\begin{array}{cc} \text{VBV}({\text{dCBV}}_{c}/\text{dt})= & \text{QFT}({\text{CFT}}_{c}/{\text{RFT}}_{c})\\ & +\text{QBN}({\text{CBN}}_{c}/{\text{RBN}}_{c})\\ & +\text{QHR}({\text{CHR}}_{c}/{\text{RHR}}_{c})\\ & +\text{QKD}({\text{CKD}}_{c}/{\text{RKD}}_{c})\\ & +\text{QMS}({\text{CMS}}_{c}/{\text{RMS}}_{c})+\text{QSK}\\ & *\left({\text{CSK}}_{c}/{\text{RSK}}_{c}\right)+\text{QLV}\left({\text{CLV}}_{c}/{\text{RLV}}_{c}\right)\\ & +\text{QBR}({\text{CBR}}_{c}/{\text{RBR}}_{c})\\ & +\text{QRB}\left({\text{CRB}}_{c}/{\text{RRB}}_{c}\right)-\left(\text{QC}\right){\mathrm{CBV}}_{c} \end{array}$$

Mixed venous plasma:

(A10) $${\text{CPV}}_{c}={\text{CBV}}_{c}/\text{BLPLR}$$
